# Comprehensive evaluation of antibacterial activity, pH stability, and surface characterization of a mussel—inspired polymer for root perforation repair

**DOI:** 10.3389/fdmed.2026.1802925

**Published:** 2026-03-30

**Authors:** Lakshmi Nidhi Rao, Aditya Shetty, Veena Shetty, Heeresh Shetty

**Affiliations:** 1 Department of Conservative Dentistry and Endodontics, NITTE (Deemed to be University), AB Shetty Memorial Institute of Dental Sciences, Mangaluru, India; 2 Department of Microbiology, K S Hegde Medical Academy, NITTE (Deemed to be University), Mangaluru, India; 3 Department of Conservative Dentistry and Endodontics, Nair Hospital Dental College, Mumbai, Maharashtra, India

**Keywords:** antibacterial activity, bioactive glass, pH, polydopamine, root perforation repair

## Abstract

Root perforation is a serious complication in endodontic therapy that can adversely affect treatment outcomes due to persistent microbial contamination and the limitations of currently available repair materials. Although mineral trioxide aggregate (MTA) is widely used, its handling characteristics, prolonged setting time, and variable antibacterial activity necessitate the development of alternative materials. This study evaluated a novel bio-inspired composite combining polydopamine, a mussel-derived adhesive polymer, with bioactive glass, designed to enhance antibacterial performance, surface characteristics, and pH behaviour relevant to root perforation repair. It was hypothesized that the experimental composite would exhibit improved antibacterial efficacy and a favourable surface nanoarchitecture compared with MTA, while maintaining physiologically relevant alkalinity. Antibacterial activity was assessed using agar diffusion against Porphyromonas endodontalis and Enterococcus faecalis, pH was measured at 24 h and 7 days, and surface characteristics were analysed using atomic force microscopy and field emission scanning electron microscopy. Antibacterial outcomes were compared using the Mann–Whitney *U* test, while pH values were analysed using independent *t*-tests. The experimental material demonstrated significantly greater antibacterial activity against Porphyromonas species (*P* < 0.05), with no significant difference against E. faecalis. A lower initial pH with gradual alkalinization and a smoother surface profile were observed. These findings suggest that the polydopamine–bioactive glass composite shows promise as an experimental material for root perforation repair, warranting further biological and translational investigation.

## Introduction

Root perforation is a clinically significant complication in endodontic therapy that may arise from iatrogenic errors, extensive caries, or resorptive defects, resulting in an unintended communication between the root canal system and the periodontal tissues (1, 2). Such defects compromise the sealing integrity of the tooth, facilitate microbial ingress, and adversely affect treatment prognosis if not effectively managed.

The success of perforation repair depends largely on the physicochemical and biological properties of the repair material. Ideally, these materials should exhibit biocompatibility, dimensional stability, resistance to dissolution, effective sealing in moist conditions, and intrinsic antibacterial activity (3). Despite continued advances in endodontic biomaterials, no material consistently satisfies all these criteria, and clinical outcomes remain variable (4).

Mineral trioxide aggregate (MTA) is widely regarded as a reference material for perforation repair due to its sealing ability, biocompatibility, radiopacity, and capacity to set in the presence of moisture (5, 6). However, its clinical use is limited by difficult handling, prolonged setting time, high cost, and inconsistent antibacterial efficacy, particularly against persistent endodontic pathogens (7, 8). These limitations have prompted the exploration of alternative materials with improved antimicrobial performance and interfacial stability.

Bioinspired material approaches have gained increasing interest in endodontics. Marine mussels adhere strongly to diverse substrates under wet conditions through catechol-mediated interactions (9, 10). Polydopamine, a synthetic analogue of this mechanism, demonstrates excellent biocompatibility, strong surface adhesion, and reported antibacterial properties, making it a promising candidate for endodontic repair applications (11).

Bioactive glass (BAG) is a well-established biomaterial capable of inducing hydroxyapatite formation, promoting hard tissue regeneration, and releasing biologically active ions in aqueous environments (12, 13). Ion release from BAG contributes to alkalinity, bioactivity, and antibacterial effects, supporting periradicular healing.

Microbial persistence remains a primary cause of endodontic failure, as bacteria and their by-products may penetrate microgaps at the tooth–material interface even after thorough chemomechanical debridement (6, 14). Consequently, repair materials with sustained antibacterial activity, favourable surface characteristics that limit bacterial adhesion, and controlled alkalinity are of particular clinical relevance (15–17).

Based on these considerations, a novel composite integrating polydopamine with bioactive glass was developed to combine wet adhesion, antibacterial activity, ion release, and surface nanoarchitectural control. Previous investigations have demonstrated favourable radiopacity and superior sealing ability of this composite compared with MTA (9). However, its antibacterial behaviour, pH dynamics, and surface characteristics require further evaluation.

Therefore, the present study aimed to assess the antibacterial activity, pH behaviour, and surface nanoarchitecture of a polydopamine–bioactive glass composite in comparison with mineral trioxide aggregate. It was hypothesized that the experimental material would exhibit enhanced antibacterial performance, a smoother surface profile, and a controlled alkaline response over time, addressing key limitations of conventional perforation repair materials.

## Materials and methods

### Synthesis of the polydopamine–bioactive glass composite

The experimental polydopamine–bioactive glass (PDA–BG) composite was synthesized under alkaline conditions to facilitate dopamine polymerization. Briefly, a 0.05 M tris (hydroxymethyl)aminomethane buffer solution was prepared in distilled water and adjusted to pH: 8.8, a prerequisite for dopamine oxidation and self-polymerization. Dopamine hydrochloride (Sigma-Aldrich; MW: 189.64 g/mol) was dissolved in the buffered solution under continuous mechanical agitation (200–250 rpm) at ambient temperature. Polymerization occurred through oxidation of the catechol moieties in the presence of dissolved oxygen.

Bioactive glass powder (Sigma-Aldrich; particle size 50–250 μm) was incorporated at 10% w/w, followed by the addition of barium sulfate (10% w/w) to impart radiopacity and calcium chloride (10% w/w) to enhance hydration kinetics and reduce setting time. The reaction mixture was stirred continuously for 24 h, filtered using Whatman filter paper, and vacuum-dried overnight to obtain the final composite powder.

### Ethical approval

Ethical clearance for the experimental protocol was obtained from the Central Ethics Committee of Nitte (Deemed to be University) (Ethics/NU/CEC/I/2021/124).

### Experimental groups

Based on a pilot assessment, the effect size was calculated to be 0.991, alpha error rate at 5%, power was set at 80%. Using these parameters the sample size was calculated as 16 specimens per group. The materials evaluated were:

- **Group 1:** Experimental polydopamine–bioactive glass composite
- **Group 2:** Mineral trioxide aggregate (ProRoot MTA)

### Surface characterization

#### Atomic force microscopy

Surface nanoarchitecture and roughness parameters were evaluated using a Flex-Axiom atomic force microscope (Nanosurf, Switzerland). Specimens were analysed in tapping mode to avoid surface damage. Root mean square (RMS) roughness (Sq) and average roughness (Sa) values were obtained using Gwyddion software for quantitative surface analysis.

#### Field emission scanning electron microscopy and elemental analysis

Surface morphology was examined using a field emission scanning electron microscope (Gemini SEM 300, Carl Zeiss, Germany). Prior to imaging, specimens were sputter-coated with gold for 15 min to minimize charging artifacts. Energy-dispersive x-ray spectroscopy (EDAX) was performed to determine the elemental composition of the composite and confirm successful material integration.

### Antibacterial activity

Antibacterial activity was assessed using the agar diffusion method against *Enterococcus faecalis* and *Porphyromonas* endodontalis. Standardized bacterial suspensions were prepared in brain heart infusion broth and adjusted to the 0.5 McFarland standard (∼1 × 10^7^ CFU/mL). Sterilized material discs (10 mm diameter × 3 mm thickness) were placed on inoculated agar plates and incubated at 37 °C under aerobic conditions for *E. faecalis* and anaerobic conditions for *Porphyromonas*. Inhibition zones were measured at three equidistant points using a digital caliper, and the mean inhibition diameter was calculated after subtracting the specimen diameter.

### pH analysis

Polyethylene tubes (3 mm diameter × 1 mm length) were filled with freshly prepared materials and immersed in 10 mL of deionized water. pH measurements were recorded at 24 h and 7 days using a calibrated digital pH meter (Merck Life Science Pvt Ltd.), standardized with pH 7.0 buffer solutions prior to measurement.

### Statistical analysis

Data were analysed using appropriate parametric or non-parametric tests based on distribution normality. Antibacterial outcomes were compared using the Mann–Whitney *U* test, while pH values were analysed using independent *t*-tests. Statistical significance was set at *P* < 0.05. Shapiro–Wilk test was used to check for normality.

## Results

### Surface morphology and elemental composition

**Figure 1:**
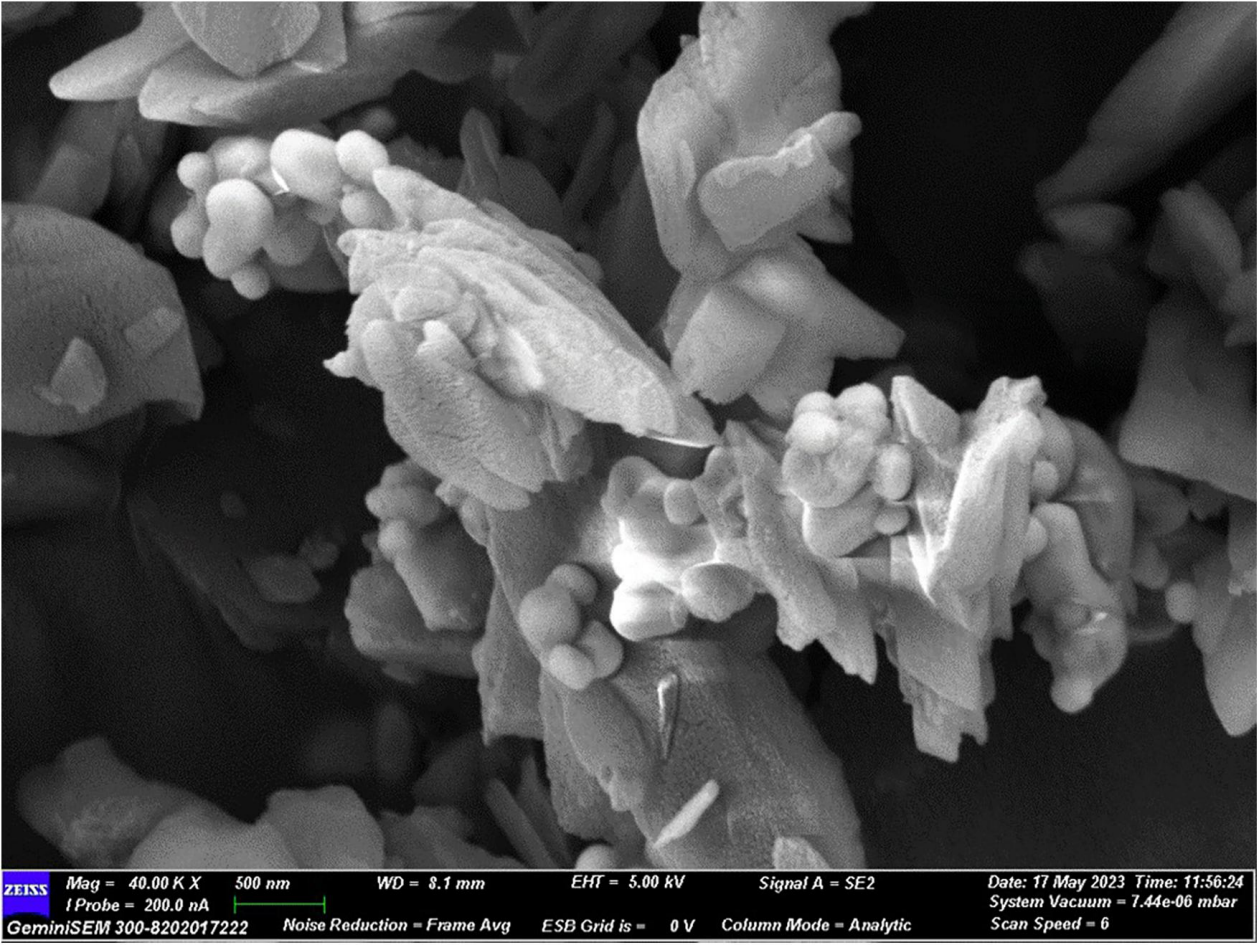
SEM image of the PDA- BAG composite with 40,000× magnification.

Field emission scanning electron microscopy demonstrated successful formation of a composite structure in the experimental polydopamine–bioactive glass material. The micrographs revealed flake-like bioactive glass particles and spheroidal barium sulfate particles uniformly embedded within a continuous polydopamine matrix (Figure 1). This morphology indicates effective polymerization and coating rather than simple physical admixture of constituents.

**Figure 2:**
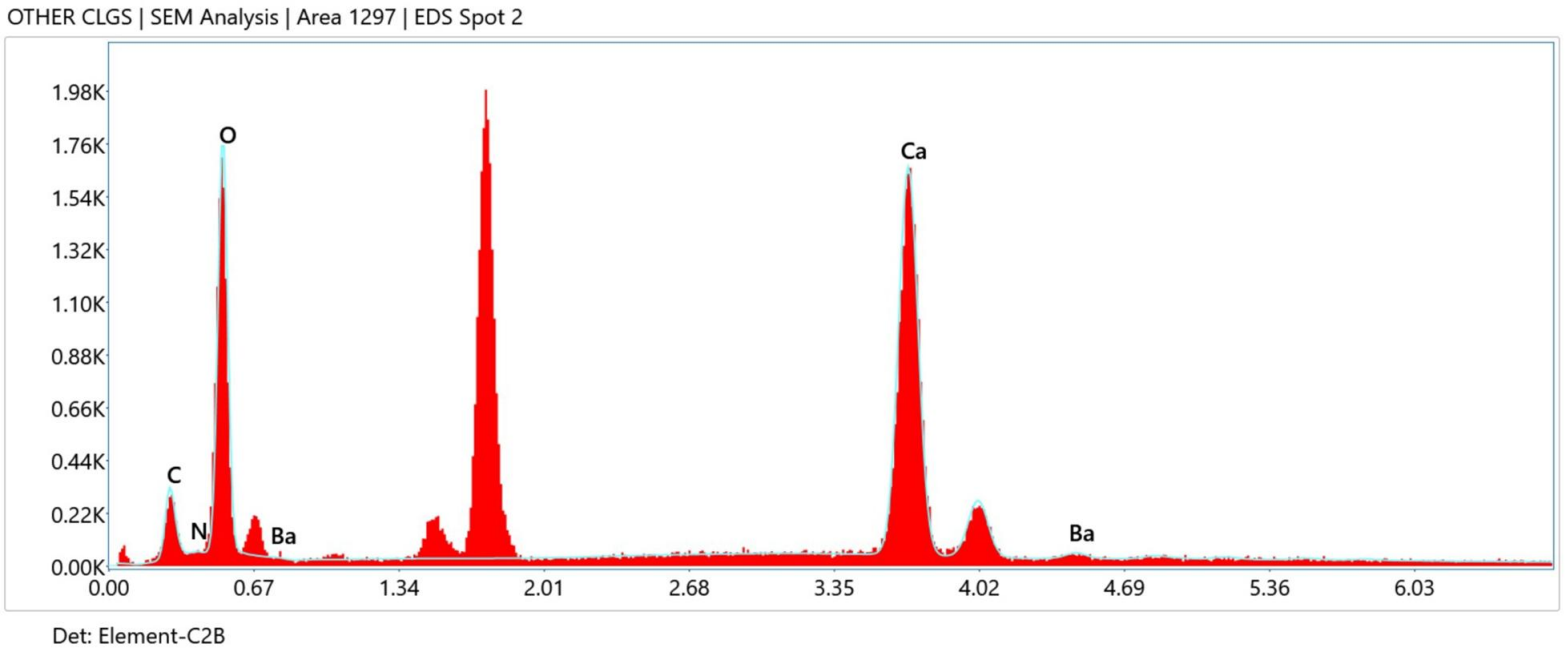
Energy-dispersive x-ray analysis.

Energy-dispersive x-ray analysis confirmed the presence of calcium, oxygen, carbon, nitrogen, and barium, corresponding to the components used in material synthesis (Figure 2). The relative enrichment of carbon and nitrogen supported the formation of a polydopamine matrix, while calcium-rich regions reflected incorporation of bioactive glass. As elemental analysis served a confirmatory purpose, quantitative tabulation was not emphasized.

### Surface nanoarchitecture

**Figure 3:**
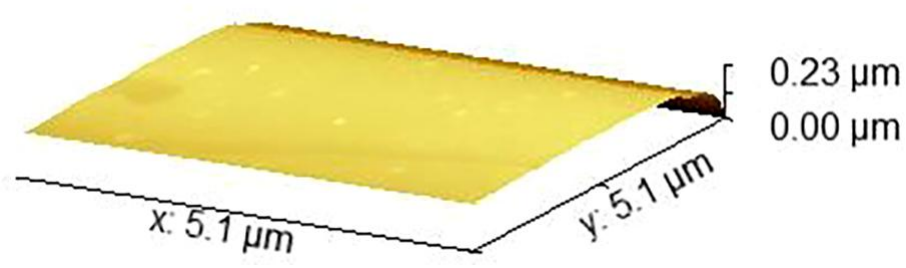
AFM image of the PDA-BAG composite.

**Figure 4:**
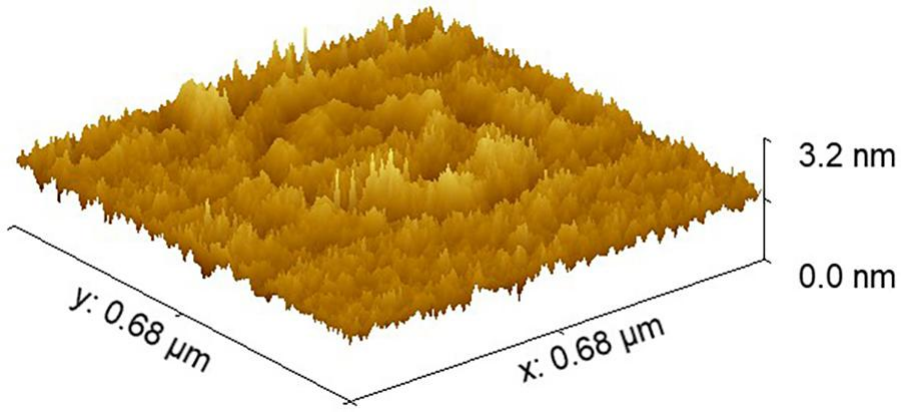
The AFM image of MTA.

Atomic force microscopy revealed a pronounced difference in surface roughness between the experimental material and mineral trioxide aggregate. The polydopamine–bioactive glass composite exhibited a root mean square roughness of approximately 1.38 nm, whereas MTA demonstrated a substantially higher roughness of approximately 1.86 nm (Figures 3, 4).

This nearly order-of-magnitude reduction in surface roughness indicates a markedly smoother nanoarchitecture for the experimental material. From a biological perspective, smoother surfaces at the sub-nanometer scale are associated with reduced bacterial adhesion and more uniform protein adsorption, whereas increased nanoscale roughness may enhance bacterial anchorage and early biofilm formation. These findings suggest that the surface characteristics of the experimental material may be more favorable for limiting microbial colonization at the material–tissue interface.

### Antibacterial activity

Against *Porphyromonas* species, the experimental polydopamine–bioactive glass composite demonstrated significantly greater antibacterial activity than MTA. Median inhibition zone diameters were approximately threefold higher for the experimental material, with non-overlapping interquartile ranges between groups, indicating a consistent antibacterial advantage (Table 1). This difference was statistically significant (*P* < 0.001).

**Table 1:** Antibacterial activity against *Porphyromonas* species (agar diffusion test).

Group	Median inhibition zone (mm)	IQR (25th–75th percentile)	*P* value	Statistical test
Experimental material (PDA–BG)	3.0	3.0–4.0	<0.001	Mann–Whitney *U*
Mineral trioxide aggregate (MTA)	1.0	0.0–1.0		

In contrast, antibacterial activity against *Enterococcus faecalis* did not differ significantly between the two materials (Table 2). Although the experimental material showed a higher median inhibition zone diameter, the interquartile ranges overlapped and the difference did not reach statistical significance (*P* = 0.078), indicating comparable antibacterial performance against this resistant facultative anaerobe.

**Table 2:** Antibacterial activity against *Enterococcus faecalis* (agar diffusion test).

Group	Median inhibition zone (mm)	IQR (25th–75th percentile)	*P* value	Statistical test
Experimental material (PDA–BG)	1.75	1.00–2.00	0.078	Mann–Whitney *U*
Mineral trioxide aggregate (MTA)	1.00	0.75–1.25		

### pH behaviour

At 24 h, the experimental material exhibited a significantly lower pH compared with MTA (Table 3). Over time, a progressive increase in pH was observed for the experimental material. By 7 days, no statistically significant difference in pH was detected between the two materials, indicating convergence of alkalinity profiles (Table 3).

**Table 3:** pH values of the experimental material and MTA at 24 h and 7 days.

Time point	Group	Mean pH ± SD	95% CI of the difference	*P* value
24 h	Experimental material (PDA–BG)	8.07 ± 0.16	−3.98 to −3.69	<0.001
	Mineral trioxide aggregate (MTA)	11.91 ± 0.23		
7 days	Experimental material (PDA–BG)	9.59 ± 0.19	−0.17 to 0.12	0.719
	Mineral trioxide aggregate (MTA)	9.62 ± 0.19		

This pattern reflects a delayed but sustained alkalinization for the experimental composite, in contrast to the high initial alkalinity observed with MTA.

## Discussion

Root perforation represents a clinically significant complication in endodontic therapy, often compromising treatment outcomes due to persistent microbial contamination and the challenges associated with achieving an effective seal between the root canal system and the periodontal tissues (1–4). As emphasized in previous literature, the prognosis of perforation repair is highly dependent on the physicochemical and biological properties of the repair material, particularly its sealing ability, biocompatibility, and antibacterial activity (4–8). In this context, the present study evaluated a polydopamine–bioactive glass composite with the aim of addressing limitations associated with conventional perforation repair materials, using mineral trioxide aggregate as the reference standard and considering current developments in bioactive repair cements (4–8, 17).

A principal finding of this investigation was the significantly greater antibacterial activity of the experimental composite against *Porphyromonas* species compared with MTA. Anaerobic Gram-negative bacteria, including *Porphyromonas*, are frequently associated with persistent endodontic infections and treatment failure due to their ability to survive in low-oxygen environments and resist conventional chemomechanical disinfection protocols (14). The enhanced antibacterial effect observed in the present study is likely attributable to the synergistic interaction between polydopamine and bioactive glass. Polydopamine, inspired by mussel adhesive proteins, contains catechol and amine functional groups capable of interacting with bacterial cell membranes, resulting in membrane destabilization and impaired cellular function (18). In parallel, bioactive glass contributes through the release of calcium and silica ions, which generate localized ionic imbalance and alkalinity that are unfavourable for bacterial survival (15, 16, 19, 20). The consistency of inhibition zone measurements suggests that this antibacterial effect is intrinsic to the composite formulation rather than a transient or diffusion-dependent phenomenon.

In contrast, antibacterial activity against *Enterococcus faecalis* did not differ significantly between the experimental material and MTA. This observation is clinically relevant, as *E. faecalis* is well recognized for its exceptional resistance to alkaline environments, ability to penetrate dentinal tubules, and capacity to survive under nutrient-deprived conditions (21–24). The comparable performance of both materials against this pathogen indicates that the antibacterial advantage of the experimental composite may be species-specific rather than universal. Importantly, the absence of statistical significance does not imply inferior performance, but rather suggests equivalence in antibacterial efficacy against a highly resilient microorganism commonly implicated in endodontic failure.

Surface nanoarchitecture is another critical determinant of the biological performance of endodontic repair materials, influencing bacterial adhesion, protein adsorption, and host cell interactions at the material–tissue interface (25, 26). Atomic force microscopy revealed that the polydopamine–bioactive glass composite exhibited a surface roughness nearly an order of magnitude lower than that of MTA. At the nanoscale, smoother surfaces are associated with reduced bacterial adhesion due to decreased surface area and fewer topographical niches for microbial anchorage (27). Moreover, smoother nanoarchitectures promote more uniform protein adsorption, which may facilitate favourable cell–material interactions and tissue integration. In contrast, increased nanoscale roughness, as observed with MTA, can enhance bacterial retention and early biofilm formation (25, 28). From a biological standpoint, the substantially smoother surface of the experimental composite represents a clear advantage in limiting microbial colonization following perforation repair.

The pH behaviour of the experimental material further differentiates it from MTA. While MTA exhibited a markedly high initial pH at 24 h, the experimental composite demonstrated a more moderate alkalinity that increased gradually over time, converging with MTA by 7 days. The importance of pH in endodontic repair materials has been well documented, as alkalinity influences both antibacterial activity and tissue response (29, 30). Excessively high initial pH values have been associated with cytotoxic effects and may negatively affect early cellular viability (31, 32). In contrast, a gradual and sustained increase in alkalinity may support antimicrobial effects while maintaining a more favourable environment for periradicular healing and mineralization (33, 34).

The physicochemical behaviour observed in the present study should be interpreted alongside previously reported findings on the same material system. Earlier investigations have demonstrated favourable radiopacity, superior sealing ability, comparable biocompatibility, and sustained calcium ion release for the polydopamine–bioactive glass composite (9, 35). Sustained calcium ion release is particularly relevant, as calcium ions play a key role in hydroxyapatite formation and have been shown to support the differentiation of dental pulp–derived cells, contributing to biological sealing and tissue repair (36, 37). Collectively, these properties indicate that the experimental composite addresses several essential requirements for an ideal perforation repair material through a multi-mechanistic mode of action.

### Limitations

Several limitations of the present study should be acknowledged. Antibacterial activity was evaluated using the agar diffusion method, which primarily reflects diffusible antibacterial effects and may not fully replicate clinical conditions characterized by direct material–bacteria contact and biofilm formation. In addition, the investigation was conducted under static *in vitro* conditions and did not include long-term aging, biofilm-based antibacterial models, or *in vivo* validation. Sample size was calculated based on pilot study as there was no previous literature to estimate the effect size. Because of the limited sample size of the pilot study the variability may be imprecise which might influence the final power of the study.

These limitations restrict direct clinical extrapolation and underscore the need for further experimental and translational studies.

### Clinical and research implications

Within these limitations, the findings suggest that the polydopamine–bioactive glass composite exhibits a combination of antibacterial efficacy, surface characteristics, and pH behaviour that is biologically and clinically relevant for root perforation repair. Future investigations should include biofilm-based antibacterial models, long-term physicochemical stability assessments, and *in vivo* validation, particularly in comparison with newer bioactive repair materials.

## Conclusion

Within the limitations of this *in vitro* study, the polydopamine–bioactive glass composite demonstrated favourable antibacterial activity against anaerobic pathogens, comparable efficacy against *Enterococcus faecalis*, a smoother surface nanoarchitecture, and a controlled alkalinization profile when compared with mineral trioxide aggregate. These properties are relevant to the biological requirements of root perforation repair, where microbial control and interfacial stability are critical.

Taken together with previously reported findings on sealing ability, radiopacity, biocompatibility, and calcium ion release, the experimental material shows potential as a promising bio-inspired candidate for perforation repair. Further biofilm-based and *in vivo* investigations are required to establish its long-term clinical applicability.

## Data Availability

The original contributions presented in the study are included in the article/Supplementary Material, further inquiries can be directed to the corresponding author/s.
